# 3D designed in-house positioning and cutting guide system for genioplasty surgery. Technical note and case report

**DOI:** 10.4317/jced.60896

**Published:** 2023-10-01

**Authors:** Íñigo Aragón-Niño, Álvaro-Damián Moreiras-Sanchez, José-Luis del Castillo-Pardo de Vera, Marta-María Pampín-Martinez, María Barajas-Blanco, José-Luis Cebrián-Carretero

**Affiliations:** 1 Medical Resident. Oral and Maxillofacial Surgery Department. La Paz University Hospital. Madrid, Spain; 2 Physician attending / Faculty. Oral and Maxillofacial Surgery Department. La Paz University Hospital. Madrid, Spain; 3 Physician attending / Faculty. Oral and Maxillofacial Surgery Department. Toledo University Hospital. Toledo, Spain; 4 Head of the Department. Oral and Maxillofacial Surgery Department. La Paz University Hospital. Madrid, Spain

## Abstract

Advances in 3D printing technology have the potential to significantly improve the workflow of maxillofacial surgical planning. In-house fabricated custom positioning and cutting guides allow for intraoperative reproduction of pre-planned osteotomy cuts, which can result in greater surgical accuracy and patient safety while maintaining an acceptable cost-effectiveness ratio. The design and creation of the customized surgical guides is performed in our hospital fab lab, which allows time savings, from an average of 10 days to just 24 hours, and a cost reduction of more than 90%. The process begins with the import of the pre-surgical facial CT scan into 3D software that allows to perform the surgical cuts virtually and the manipulation of the segments. Once the virtual planning of the surgery has been performed, the next step is the creation of the cutting and positioning guides. The final step is the printing of the guides in surgical resin and their sterilization. In addition, post-surgical models can be 3D printed to pre-mold the plates on them, which saves surgical time. The mentoplasty surgery is a simple example of how 3D surgery can be applied to maxillofacial surgery in an efficient way obtaining all the advantages of customized surgery with a limited investment in time and resources.

** Key words:**3Dsurgery, customized, personalized medicine, genioplasty, surgical guides, in house.

## Introduction

Mentoplasty is the surgical technique used to correct chin deformity. It consists of an osteotomy in the lower edge of the jaw that allows the movement of the chin in three dimensions and fixation in the new desired position. The first available publications of this surgical technique date back to 1957 (1). First through an extraoral approach by Gillies and Milard and a little later through an intraoral approach by Trauner and Obwegeser. Since then, mentoplasty surgery and facial surgery in general have come a long way.

The key to the best surgical outcome is an optimal surgical plan. In recent years the concept of 3D surgery has been developed, which allows virtual surgical planning of the location of the cuts and the desired movement prior to surgery.

3D surgery allows us to move away from on-site planning of the surgery to pre-surgical planning of the desired movement, with a simulation and preview of the result at the bone and soft tissue level. Surgical guides allow us to transfer the information from the virtual planning to the operating room, enabling surgeons to carry out the surgical plan in the OR (2).

Surgical guides are patient customized devices that allow the surgeon to carry out the surgical plan in the surgery field. The cutting guides allow the surgeon to guide the point of the cut in the bone to avoid damage of important structures. The positioning guides allow the surgeon to bring the segment to the desired and previously planned position with a millimeter level of accuracy (3).

Advances in 3D printing technology have the potential to significantly improve the workflow of maxillofacial surgical planning. In house printed customized positioning and cutting guides allow for intraoperative reproduction of pre-planned osteotomy cuts, which can result in greater surgical accuracy and patient safety while maintaining an accepTable cost-effectiveness ratio (3-7).

Case Report

The first step is to interview and explore the patient to determine his or her needs and goals. Within the physical exploration, a fundamental step is the facial analysis which allows us to determine the anthropometric measurements of the face and analyze the required corrections. In addition, a facial CT scan is requested to be able to perform the surgical planning.

1. Virtual surgical planning:

Once we have the information from the facial analysis and the facial CT scan, we move on to virtual planning. For virtual planning, Dolphin Imaging® software (Patterson Dental, Saint Paul, Minnesota, USA) is used. Alternative free options are available. With the CT image file and scan data, we plan and simulate the surgery according to the required needs. It allows to see the pre and post results both at the bone and soft tissue level. We design the cutting lines on the bone for the type of movement we want to achieve.

**Figure 1:**
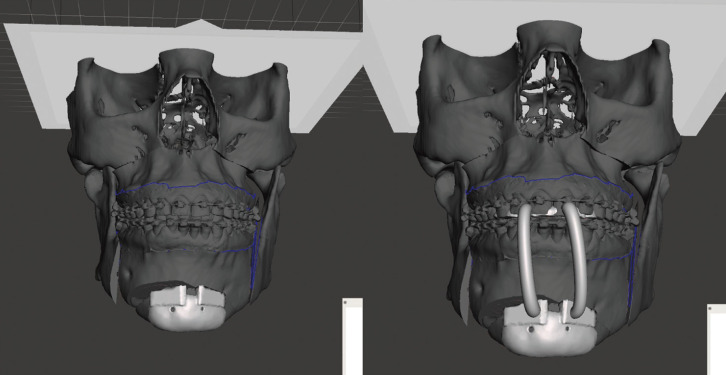
Surgical guide design.

Once we have the pre and post surgical models, we take them to a 3D file editing software. In our case we use Meshmixer® (v 3.5, Autodesk Inc., San Rafael, CA, USA). We use the pre-surgical model to generate the cutting guide and the post-surgical model to generate the positioning guide, (Fig. 1).

2. 3D printing:

The designed guides are exported in STL format for the 3D printing stage. The digital models are fabricated using a stereolithography (SLA) 3D printer (Form 2, Formlabs, Somerville, MA, USA) and Surgical Guide resin (Formlabs) with a printing resolution of 0.1 mm. After printing, the guides undergo a cleaning process in a Form Wash (Formlabs) containing 99% isopropyl alcohol for 20 minutes to remove excess liquid resin. They are subsequently subjected to post-curing at 60 °C for 30 minutes in a Form Cure (Formlabs) to ensure biocompatibility and optimal mechanical properties. Finally, the models are sterilized and sent to the operating room.

We can also export and print a post-surgical model of the chin on which we can pre-fold the surgical plates to save surgical time. The printing time is four hours and 23 minutes, washing and curing time is approximately 40 minutes, therefore printing and post processing time is around 5 hours to have the in house guides ready to send for sterilization.

Regarding the cost, the total amount of resin required for this case is less than 70ml. The price of 1 liter of biocompatible resin suiTable for surgical use is 225€, so the approximate cost for this case is 15€.

3. Surgical case:

The first step is to perform the usual intraoral approach for mentoplasty surgery.

**Figure 2:**
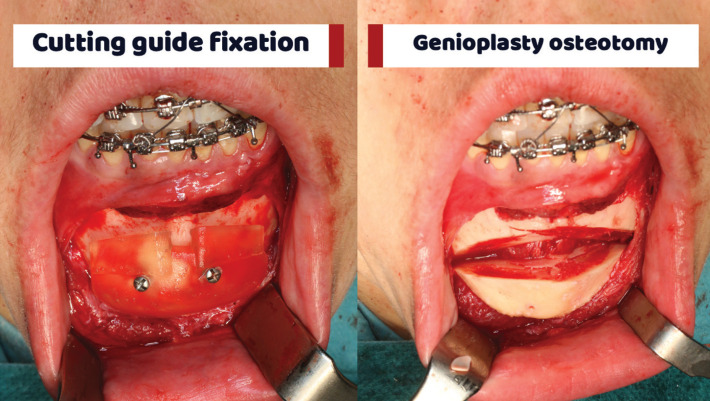
Cutting guide surgical use and osteotomy.

Once the chin is exposed, the customized cutting guide is attached. It is perfectly adapted to the bone contour because it has been designed on the 3D model of the surgical planning with the CT images. Using this guide, the chin osteotomy is performed, (Fig. 2).

**Figure 3:**
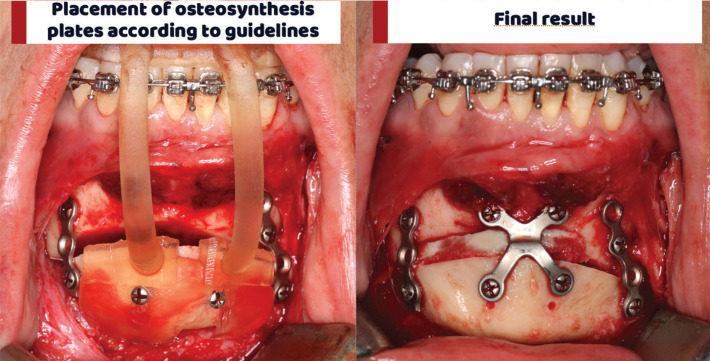
Positioning guide surgical use and plate fixation.

Once the osteotomy has been performed, the positioning guide is placed to move the distal segment to the desired and previously planned position. Keeping the positioning guide, we place the lateral osteosynthesis plates and finally the central one, (Fig. 3).

Note that these plates can be premolded on the final 3D printed model of the chin to save surgical time.

## Discussion

The two main advantages of using self-made cutting guides are cost savings and production time savings. We have the high initial cost of the necessary equipment, the learning curve in the use of the necessary programs, and the time spent in the design to contend with.

A custom design plate and cutting guides for mentoplasty performed by a commercial company cost approximately € 1500 and have an average delivery time of 7 to 10 days including the design, planning, and production phases (7).

With in-house cutting guides, the total cost is reduced to around € 100 per case. The cost of the plates and screws should be added. In addition, the design and production times are less than 48 hours.

The use of in-house cutting guides seems a reliable and recommended alternative in mentoplasty surgery. It allows to determine the cuts with greater precision and in a predictable way to the surgical planning. It is also a cost-effective option for saving surgical time.

The learning curve for developing expertise with planning software and printing settings is compensated by increased surgical predictability and decreased operating time, making this type of planning a worthwhile investment.

We can therefore say that the use of self-created guides has some advantages, among which the following stand out:

• Less dependency on industry.

• Less need for time margin, which allows the patient to be operated on earlier.
